# Verses

**Published:** 1907-08

**Authors:** 


### VERSES.

### LET SOMETHING GOOD BE SAID.

When over the fame of friend or foe
The shadow of the grave shall fall; instead
Of words of blame or proof of thus and so,
Let something good be said.

—Riley.

### IF HORSES COULD TALK.

Somebody wishes that horses could talk.
Explaining the causes that force them to balk.
The wish is a good one, but just let us hint
Their views about autos we never could print.

—Cleveland Plain Dealer.

### GOING UP.

"Heaven is not reached at a single bound,
But we build the ladder by which we rise
From the lowly earth to the vaulted skies,
And we mount the summit round by round."
"We rise by the things that are under our feet,
By what we have mastered of greed and gain,
By the pride deposed and the passion slain,
And the vanquished ills that we hourly meet."

—Exchange.

### AN ACHING TOOTH.

An aching tooth "disturbed his peace;"
Said he, "Egad! forsooth,
I'll go swear out a warrant
And the cops will 'pull' the tooth."

—Office and Laboratory.

### MAN AND WOMAN.

What meks er dog bark de moon,
Kose der'es er man een hit,
Wot meks him howl er chune,
He lows trubble's comin by hit.

Dats de way wid ebry ting,
Der'es er reason wy for hit,
Wedder we cry, cuss er sing,
Der'es allers sumfin fer hit.

De man he allers sees hit fuss,
Den lites out ter cus hit,
De woman lows dat cum it mus,
Den sets out to cry an fuss hit.

—B. H. Teague.

